# Immunoglobulin G Determination in Human Serum and Milk Using an Immunosensor of New Conception Fitted with an Enzyme Probe as Transducer

**DOI:** 10.3390/s8106727

**Published:** 2008-10-28

**Authors:** Luigi Campanella, Dalina Lelo, Elisabetta Martini, Mauro Tomassetti

**Affiliations:** Department of Chemistry, University of Rome “La Sapienza”, P.le A. Moro 5, 00185 Rome, Italy; E-mail: luigi.campanella@uniroma1.it

**Keywords:** Immunosensor, enzymatic transducer, Immunoglobulin G detection, human milk, human serum, urea interference

## Abstract

To completely overcome the problem of the presence of urea in the serum, which can be the cause (especially at low immunoglobulin G concentrations) of a small but non negligible interference in the enzyme reaction of the enzymatic marker, when the measurement was performed by a potentiometric immunosensor that we constructed and characterized in previous work, and which used urease as marker, we have now constructed an entirely different and highly innovative immunosensor. This new device uses the enzyme alkaline phosphatase as marker, sodium phenylphosphate as substrate but above all, a tyrosinase biosensor obtained by coupling a Clark type gas diffusion amperometric electrode and the tyrosinase enzyme, immobilized in a cellulose triacetate membrane, as transducer. After optimizing the ‘competitive’ measurement procedures, the new immunosensor was used to determine both HIgG and the anti-HIgG, with a limit of detection (LOD) of the order of 3×10^-11^ M. Clearly this highly innovative construction geometry makes the immunosensor extremely selective. This makes it possible to determine immunoglobulin G both in human serum and milk without the slightest interference by any urea present in these biological matrixes.

## 1. Introduction

Immunoglobulins are glycoproteins that function as antibodies. They are found in the blood and tissue fluids, as well as in many secretions. Structurally they are globulins (in the γ-region of protein electrophoresis). They are synthesized and secreted by plasma cells that are derived from the immune system B cells. There are five types of immunoglobulin, including the well known HIgG. The antibodies of immunoglobulins have two primary functions: i) they bind antigens; ii) they combine with different immunoglobulin receptors specific to them and perform effector functions. Immunoglobulin G determination is of considerable bioclinical interest as these antibodies perform the function of immune defence by removing substances extraneous to the organism [1-4]. The antibody reactions contribute substantially to the development of routine diagnostic tests for immunoglobulin G determination, which is frequently used in clinical analysis. On the other hand, a number of proteins found in milk, including HIgG, under various conditions exhibit antimicrobial activity. In particular, immunoglobulin G antibodies are protective proteins that are important in the transfer of passive immunity from the mother to the neonate.

In the last few years we developed several potentiometric immunosystems using urease as marker for the measurement of both HIgG and anti-HIgG (the determination of the latter can indeed also be useful for monitoring antibody production in the test animals) [5-7]. In previous research [7] these systems were used to determine immunoglobulin G in human serum [7]. In the present research a new immunosensor was developed that does not suffer interference from other analytes present in the serum, particularly urea, since we chose an entirely different construction geometry, i.e. alkaline phosphatase as marker and sodium phenylphosphate as substrate of the enzyme reaction, and finally a tyrosinase enzyme sensor as transducer, which makes the new immunosensor extremely selective. It thus becomes possible to determine immunoglobulin G both in human serum and in human milk samples, without any problems at all.

## 2. Experimental Section

### 2.1 Materials

The Pall-Biodyne C membranes (Nylon 6.6, porosity 0.45 μm), with carboxyl groups on the surface, were from Pall Italia S.R.L. (Milan); phenol, dialysis membrane (art. D-9777), formic acid, cellulose triacetate (TAC), Albumin (from bovine serum) (BSA) urea and TRIS (hydroxymethyl-aminomethane), TWEEN® 20 were from Sigma Aldrich srl (Milan); Monoclonal Anti-human Immunoglobulin G (catalogue number 13382-1MG), Human Immunoglobulin G from human serum (catalogue number I-5256), and Anti-human Immunoglobulin G – alkaline phosphatase (catalogue number A-9544), were from Sigma Immunochemicals (Milan); tyrosinase (EC. 1.14.18.1) extract from mushroom 3216 U mg^-1^ was from Fluka (Milan); Ny+ Immobilon Affinity membrane (a positively charged nylon membrane with polyester reinforcement optimized for reliable and reproducible transfer, immobilization, hybridization, and subsequent reprobing, porosity 0.65 μm) was from Millipore Corporation (NY); magnesium chloride, potassium phosphate monobasic, potassium phosphate bibasic and all other solvents or reagents of the highest purity were from Carlo Erba, (Milan).

### 2.2 Samples

Human serum (aseptically filled) (catalogue number S-07023, 50 mL), was purchased from Sigma Aldrich srl (Milan); Human milk samples were taken from one healthy mother in the eighth month after the birth.

### 2.3 Apparatus

The amperometric measurements were performed in a 25 mL thermostated glass cell kept under constant stirring. The Clark electrode was supplied by Amel (mod. 332) (Milan, Italy) and the amperometric measures were performed using an oximeter (Amel mod. 360) connected to a recorder (AMEL mod. 868).

## 3. Methods

### 3.1 Construction of tyrosinase biosensor

**Figure 1.**
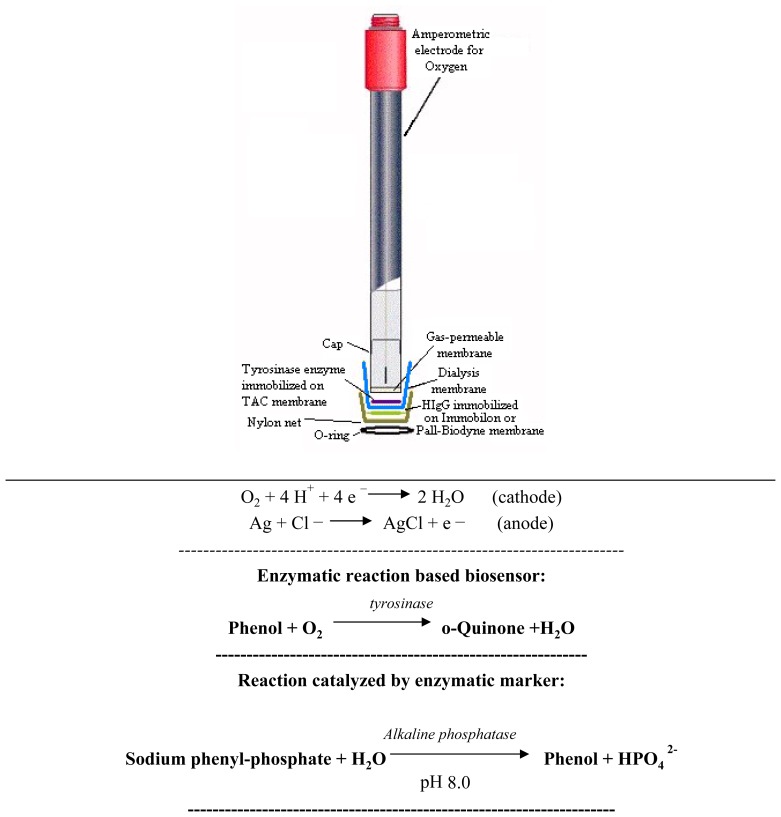
New immunosensor assembly.

The tyrosinase biosensor was composed of an oxygen amperometric electrode coupled to the tyrosinase enzyme (Figure 1), immobilized in TAC [8], or Pall-Biodyne, or Immobilon membrane [5, 7] and based on the following enzymatic reaction:

$$\text{Phenol}+{\text{O}}_{2}\overset{\text{tyrosinase}}{⟶}\;{\text{o}‐\text{Quinone}+\text{H}}_{2}\text{O}$$

Three different methods of enzymatic immobilization were tested for the purpose of constructing the tyrosinase biosensor. In the first method, enzyme immobilization was performed using a TAC membrane. In the second method, immobilization was achieved using a Pall-Biodyne membrane and in the third method an Immobilon membrane was used.

### 3.2 Immobilization of tyrosinase in TAC membrane

In practice, a cellulose triacetate viscose was prepared [8] by dissolving cellulose triacetate (4 g) in a solution of formic acid (98% w/w,) and water (90+10, v+v, 100 mL), stirring until complete dissolution was obtained (about 6 h): the cellulose triacetate viscose was rapidly extruded into a 500 μm thick film. This film was immersed in distilled water, where coagulation took place. The gel-like membrane obtained was washed repeatedly in distilled water until there were no further traces of acid in the washing water and the membrane was then preserved in distilled water until used. It was then cut into approximately 3 cm diameter disks. For enzyme immobilization, tyrosinase enzyme (3216 U mg^-1^, 5.0 mg) were weighed out and dissolved in phosphate buffer (0.1 M, 200 μL, pH 7.0). An aliquot of the enzyme solution (100 μL) was placed on one of the previously prepared TAC disks which was allowed to stand at 4°C until completely dry. Before being used, each membrane disk of TAC was rapidly rehydrated using microdrops of phosphate buffer solution. The TAC membrane, which contained the tyrosinase enzyme, was fixed between a dialysis membrane and a PTFE gas-permeable membrane on the electrode cap. The whole assembly was fixed by means of an O-ring to a cap screwed on to the head of the amperometric transducer.

### 3.3 Immobilization of tyrosinase in Pall-Biodyne membrane

The tyrosinase enzyme was chemically immobilized on a functionalized nylon membrane (Pall-Biodyne) possessing a carboxyl group on the polymer surface. The membrane was activated using a well-known procedure described in the literature [9, 10]. To this end, tyrosinase enzyme (3216 U mg^-1^, about 5.0 mg) was stratified on a Pall-Biodyne membrane disk (surface area of about 1 cm^2^) pretreated as follows: the disk was immersed in a phosphate buffer solution, 0.5 M, pH 4.8 under constant stirring. The *N*-1-ethyl-3(3-dimethylaminopropyl)carbodiimide was gradually added until a final concentration of 0.1 M was obtained. The membrane immersed in the solution was left for 40 min at room temperature under constant stirring. The membrane was then washed in phosphate buffer (pH 7), after which the tyrosinase enzyme was spread uniformly over the still damp membrane. The enzymatic membrane thus obtained was stored in a humid atmosphere at 4°C for 24 h prior to being used. The membrane thus constructed was then washed in acetate buffer 5×10^-3^ M, pH 5.6 before being fixed to the head of the oxygen electrode and covered with a dialysis membrane. Lastly the whole assembly was fixed to the electrode by means of a rubber O-ring.

### 3.4 Immobilization of tyrosinase in Immobilon Membrane

The Immobilon membrane, a positively charged nylon membrane [5], was cut into 1 cm^2^ disks and the enzyme tyrosinase solution (about 50 μL) was placed on top of the disk. The enzyme solution was prepared in an Eppendorf test tube by dissolving tyrosinase enzyme (3216 U mg^-1^, about 5.0 mg) in phosphate buffer (0.1 M, pH 7, 200 μL). The enzymatic membrane thus obtained was then placed in a humid atmosphere at 4°C, for 24 h prior to being used. The Immobilon membrane containing the tyrosinase enzyme was mounted between a dialysis membrane and a PTFE gas-permeable membrane on the amperometric electrode cap by means of a rubber O-ring.

### 3.5 Optimization of the enzyme immobilization method

A series of measures were performed to determine the best membrane to be used for the enzyme immobilization; to this end, a series of trials were carried out each time, using the tyrosinase biosensor fabricated using one of the three different membranes considered in sections 3.2-3.4; the biosensor was dipped in a thermostated cell containing phosphate buffer solution (0.1 M, pH 7, 5 mL) in which the phenol concentration was increased by the addition of 0.01 M of phenol solution (specific substrate for the enzyme, 0.5 mL), waiting for the signal to stabilize between two successive additions. The oxygen variation values (recorded as ppm after each signal stabilization) have been reported as a function of increasing phenol concentration. Straight lines were obtained successively using each of the three different enzyme membranes considered, the slope of which was calculated. The method sensitivity was checked from the slope of the straight line obtained. The entire procedure was repeated for three days with each of the membranes considered; in this way it was possible to determine the trend, as a function of the time the biosensor was in use, of both linear range and calibration sensitivity of the method with increasing phenol concentrations (from 10^-8^ to 10^-2^ M) in solution, and to compare the linear range and sensitivity of the assembled tyrosinase biosensor, respectively using each of the three immobilization methods described above.

### 3.6 HIgG immobilization on Pall-Biodyne membrane

The pre-activated Pall-Biodyne membrane, having a surface area of about 1 cm^2^, was soaked under stirring for 40 min at room temperature in a 0.5 M solution of phosphate buffer (pH 4.8) containing 0.1 M of 1-ethyl-3-(3-dimethylaminopropyl)carbodiimide. The membrane was washed with a 0.1 M phosphate buffer, pH 7.4, then 25.0 μL of a 50 mg mL^-1^ HIgG solution was added to the wet membrane. The membrane was finally allowed to stand for 24 h in a humid atmosphere at 4°C.

### 3.7 HIgG immobilization on Immobilon membrane

The Immobilon Ny+ Membrane was cut into approximately 1 cm^2^ surface disks and 25.0 μL of a 50 mg mL^-1^ Human Immunoglobulin G was directly deposited on the surface of each disk. The Immobilon membrane was then dried at room temperature for about 24 h and stored at 4° C, when not used.

### 3.8 Immunosensor assembly

In practice, four membranes were mounted on the head of the electrode, in the following order: gas-permeable membrane, enzymatic membrane (i.e. the membrane with entrapped tyrosinase enzyme), dialysis membrane and Immobilon, or Pall-Biodyne membrane with HIgG immobilized on it. The membranes were kept in place by a nylon net and a rubber O-ring.

### 3.9 Determination of anti-HIgG with new immunosensor

To this end, the Immobilon or Pall-Biodyne membrane, on which Human Immunoglobulin G (HIgG) was immobilized, was fixed to the head of the gas diffusion amperometric enzyme sensor as described in section 3.8. Before measurement, the immunosensor was dipped into a Tris-HCl buffer solution, 0.1 M (pH 8.0), containing 0.05 % Tween-20 by weight and 2.5% by weight of BSA (bovine albumin was used to minimize non specific adsorption on the membrane). The Tris-HCl buffer solution 0.1 M, pH 8.0, was renewed in the cell to which the anti-HIgG to be determined was then added to the solution containing a fixed concentration of alkaline phosphatase anti-HIgG conjugated: 20 μL (2 mg mL^-1^) in 5 mL of Tris buffer. The enzyme-conjugated antibody was allowed to compete with the non-conjugated antibody in binding with the HIgG immobilized on the membrane. After washing with the same buffer solution to remove all the unbound anti-HIgG, the specific substrate of the enzyme, i.e. sodium phenyl-phosphate, was added to the renewed buffer solution in which the immunosensor was dipped, under stirring. The following enzyme reaction occurred, catalyzed by the enzymatic marker (alkaline phosphatase):

$$\text{Sodium phenyl}‐\text{phosphate}+{\text{H}}_{2}\text{O}\overset{\text{Alkaline phosphatase}}{\underset{\text{pH}\;8.0}{⟶}}\text{Phenol}+{{\text{HPO}}_{4}}^{2‐}$$

and the signal, inversely correlated with the quantity of anti-HIgG to be measured, was recorded.

**Figure 2.**
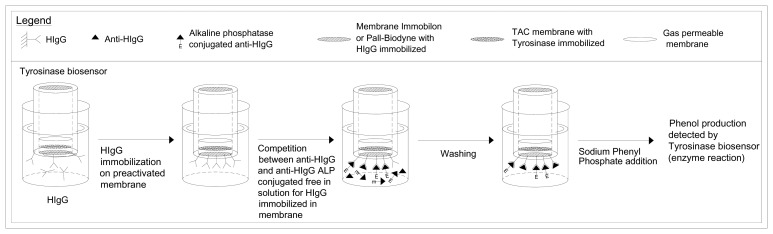
Determination of antibody (Anti-HIgG) by new immunosensor using tyrosinase enzyme electrode as transducer. Test geometry: competition for HIgG immobilized in membrane, between Anti-HIgG alkaline phosphatase conjugated and non-conjugated, both free in solution.

Lastly a calibration curve was constructed by plotting on a semilogarithmic scale the amperometric signal in parts per million of oxygen (ppm O_2_) as a function of anti-HIgG concentration in solution, used to determine the unknown concentration of anti-HIgG contained in the sample. The sequence for measuring the anti-HIgG by this method is schematized in Figure 2. The competition time was one hour while the enzymatic reaction response was about 20 minutes. The individual measures of the calibration curve were performed each time using a new membrane.

### 3.10. Determination of HIgG with new immunosensor

**Figure 3.**
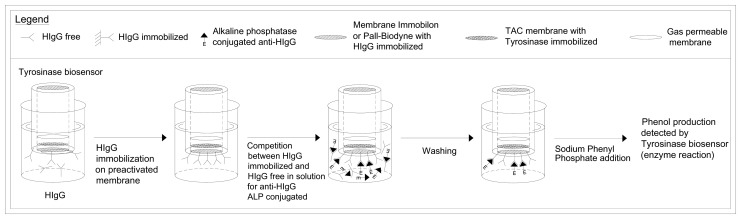
Determination of antigen (HIgG) by new immunosensor, using tyrosinase enzyme electrode as transducer. Test geometry: competition for anti-HIgG urease conjugated, between HIgG immobilized on membrane and HIgG free in solution.

The HIgG free in solution at different concentrations, or to be determined, was allowed to compete with the same antigen, but immobilized on the membrane (Immobilon, or Pall-Biodyne) overlapping the head of the amperometric electrode for oxygen, in order to produce the antibody reaction with a fixed supply of antibody, free in solution and labelled with alkaline-phosphatase. In practice, the beginning step of the procedure was the same of the previous methods reported in section 3.9. After in the cell, the Tris-HCl buffer solution, 0.1 M, pH 8.0 was renewed and a fixed concentration, i.e. 20 μL (2 mg mL^-1^) in 5 mL of Tris buffer, of the enzyme-labelled Anti-HIgG (that is, Anti-HIgG-alkaline-phosphatase conjugate) was added, then was allowed to incubate at 25° C for 1 h. The free in solution antigen (HIgG) competed with the HIgG immobilized on the membrane of the immunosensor, dipped in the same solution, in bonding the labelled anti-HIgG. On adding the enzyme substrate (phenyl-phosphate) to the renewed buffer solution, after washing with the same buffer to remove all the unlabelled Anti-HIgG not bound to the HIgG, the recorded signal was correlated with the quantity of labelled immunocomplex formed on the surface of the membrane and inversely correlated with HIgG concentration to be measured. The calibration curve obtained by plotting the current signal vs the final HIgG concentration on a semilogarithmic scale was then used to determine the concentration of the unknown Anti-HIgG. In practice the sequence of events occurring during the HIgG assay is outlined in Figure 3. The analysis times and methods used to construct the calibration curve are the same as those described in the preceding section.

### 3.11. Measures performed on the washing solutions

Finally, it was possible to construct calibration curves both for HIgG and anti-HIgG not only using the actual immunosensor itself, that is, by performing the enzymatic measures after immunosensor separation, as described in Sections 3.9 and 3.10, but also by performing the enzymatic measurement directly on the collected water (10 mL) obtained by mixing 5 mL of the solution in which the competition took place with 5 mL of the buffer washing solution used to wash the immunosensor surface once, after phenyl-phosphate addition to the latter. In this case the measurement was achieved using the simple tyrosinase biosensor. Lastly, the calibration curve was obtained by plotting the current signal vs the increasing concentration of the antigen or the antibody on a semilogarithmic scale.

### 3.12. Measures performed on the Human serum and human milk

The real samples (serum and human milk) were suitably diluted before analysis from 100 to 10,000 fold for the serum and from 100 to 1,000 fold for milk, respectively, using 0.1 M, pH 8.0 tris-HCl buffer. Five mL of this solution thus diluted was used to perform the measure as described in Section 3.10.

## 4. Results and Discussion

**Figure 4.**
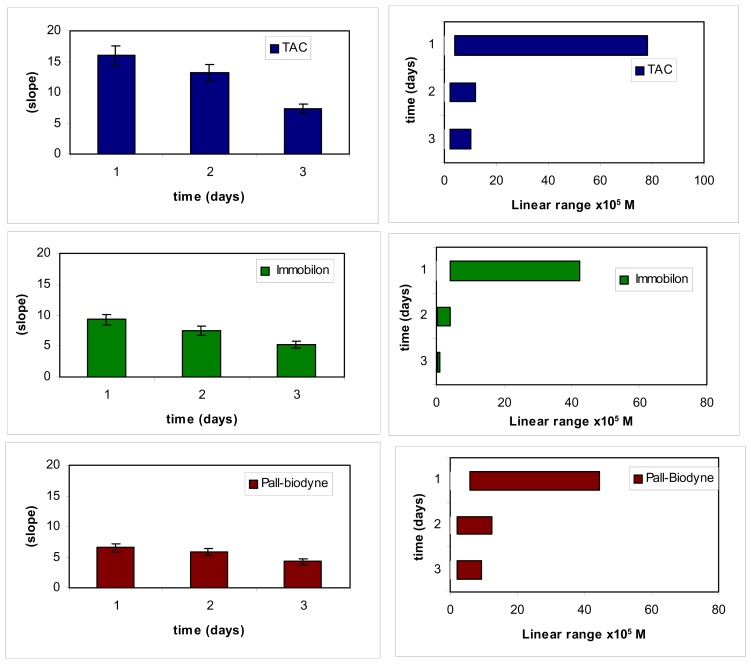
Optimization (slope and linear range during lifetime) of the membrane binding tyrosinase enzyme using three different immobilization methods, to be used for the immunoglobulin G measurement by the new immunosensor.

For the construction of the tyrosinase biosensor used as transducer for the immunosensor it was first of all necessary to optimize the enzyme immobilization method. To this end, three different methods were tried out, one of which [7] based on the entrapment of enzyme in a cellulose triacetate membrane was previously developed in our laboratory; the other two methods are the same as those used to immobilize the antigen and respectively involve, in one case, immobilization in a positively charged nylon Immobilon membrane and in the other immobilization in a pre-activated Pall-Biodyne membrane. Figure 4 shows the histograms illustrating the results obtained in several tests carried out using the three different types of immobilization over three days, starting from the time in which the immunosensor was assembled. It is observed how the calibration sensitivity (that is the slope of the calibration curves obtained) decreases from day one today three of use in all three cases; but also how in any case the sensitivity remains higher (about double) if the immobilization in TAC is used than when the other two immobilization methods are used.

**Figure 5.**
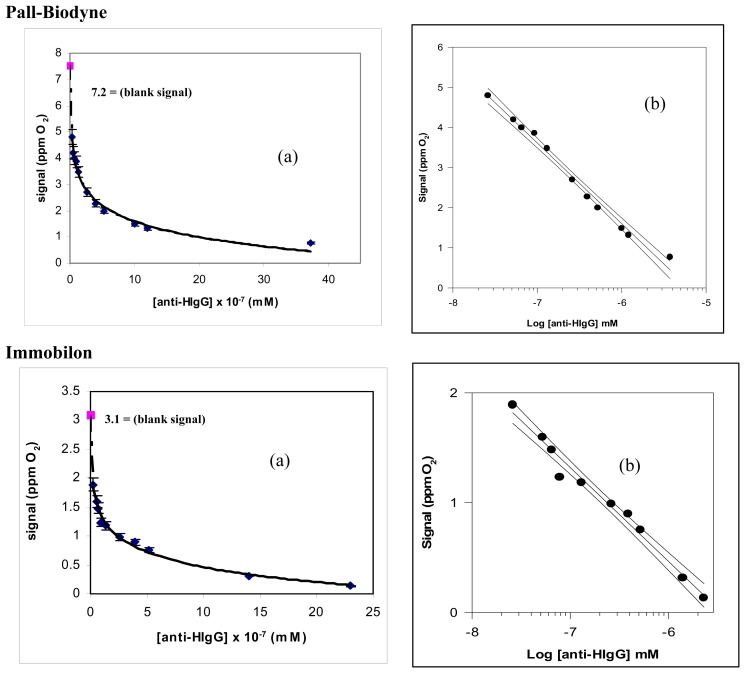
**(a)** Behaviour of the new immunosensor response as a function of growing anti-HIgG concentration, using Pall-Biodyne or Immobilon membrane; **(b)** corresponding calibration curve and confidence interval for the anti-HIgG determination, obtained using a semilogarithmic scale.

**Figure 6.**
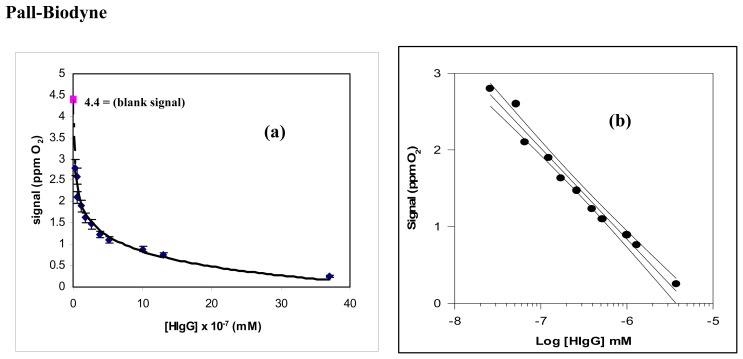

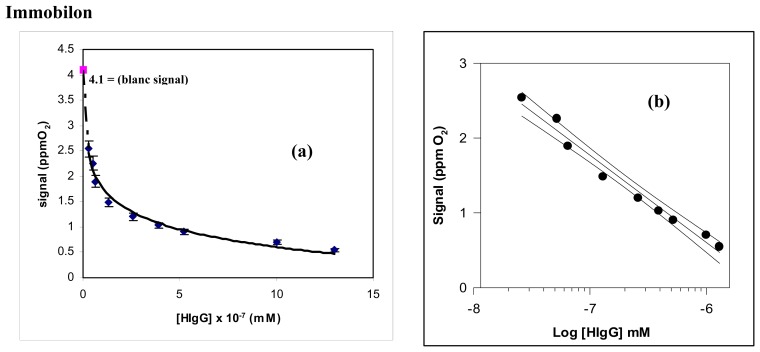
**(a)** Behaviour of the new immunosensor response as a function of growing HIgG concentration, using Pall-Biodyne or Immobilon membrane; **(b)** corresponding calibration curve and confidence interval for the HIgG determination, obtained using a semilogarithmic scale.

Consequently, in developing the new immunosensor it was decided to use immobilization of the tyrosinase enzyme in the cellulose triacetate membrane. On the other hand, as already mentioned, the antigen HIgG was immobilized both in Pall-Biodyne or Immobilon membranes, without carrying out any additional experimental tests, in view of the very good results obtained in preceding similar works [5-7] using these types of membrane for the antigen immobilization. Once the biosensor transducer had been optimized, a complete analytical characterization of the new immunosensor was performed. To this end, Figures 5 and 6 both the immunosensor responses obtained by increasing the antigen or the antibody concentration and the corresponding semilogarithmic diagrams, used as calibration curves, obtained for the determination of both anti-HIgG and HIgG, respectively, by means of the new immunosensor.

Tables 1 and 2 summarize the principal analytical data for the determination of anti-HIgG and HIgG respectively, obtained using the new immunosensor proposed in the present research. The linearity range for the immunosensor is, in any case, about two decades. The limit of detection (LOD) [11] for the new immunosensor is of the order of (0.2 - 0.3) × 10^-7^ mM, which is only about half a decade higher than those found for previously developed potentiometric immunosensors, and than that of the method with the preconcentration described in previous papers [6, 7]. With the new immunosensor immunoglobulin G detection also displays a precision (RSD% ≈ 6.6-7.4) comparable with that of the previous method using the potentiometric transducer [8].

Lastly also the correlation coefficient value of the calibration curve was comparable with that of the previous potentiometric immunosensors [7]. It is also interesting to observe that, as mentioned in section 3.11, it is possible to perform an alternative measurement of HIgG or of anti-HIgG by performing enzymatic measurement on the washing water, after competition and a separating procedure, using only the tyrosinase biosensor. Clearly, in this case, the signal will be proportional to the concentration of the alkaline phosphatase anti-HIgG conjugated, which was not immobilized in the immunosensor membrane. The recorded signal correlated directly with the antigen or antibody concentration to be measured. The results of this procedure are shown in table 3.

The data show there are no analytical benefits to be obtained using this procedure; however, the measurement can be utilized to confirm the results of the measures performed by the current immunosensor. It is clear that the immunosystem responses obtained in this case display an increasing trend that is symmetrical and opposite in sign to the decreasing trend obtained when the measure is performed directly by the new immunosensor.

**Figure 7.**
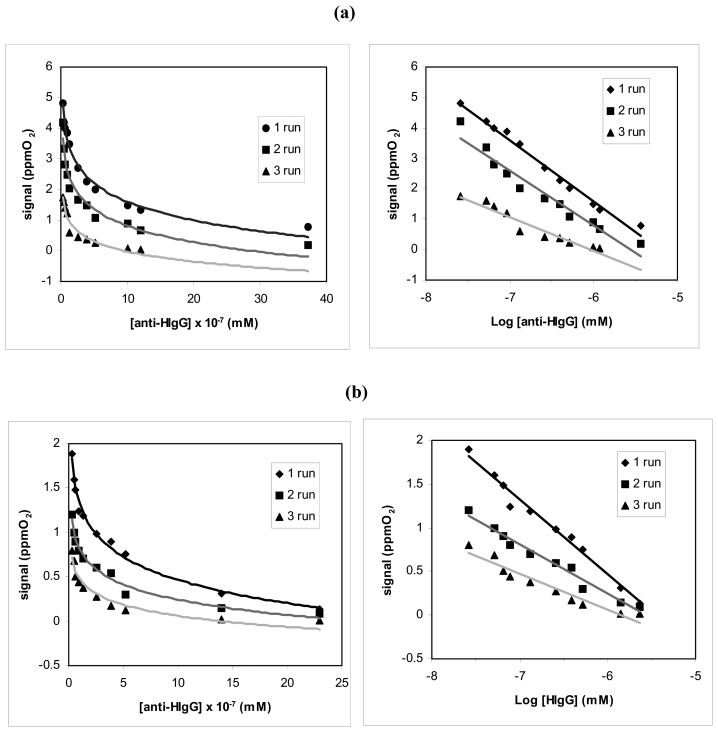
Reutilization of the immunosensor for anti-HIgG analysis after washing with glycine solution 0.1 M, at pH = 2 and MgCl_2_ 2.5 M, using **(a)** Pall-Biodyne membrane, **(b)** Immobilon membrane.

It was also attempted to optimize the method in order to allow the reutilization, albeit after only a limited number of routine measurements by immunosensor, using the Pall-Biodyne or Immobilon membrane, on the surface of which the HIgG were immobilized. In practice, the separation of the Anti-HIgG from their respective immobilized HIgG (which were combined in the antigen-antibody complex) had to be performed after each measurement, without destroying the bond between the HIgG and the polymeric membrane. The main results of this research are illustrated in Figure 7 and related table 4.

The best results have so far been obtained by gently washing with Glycine-HCl buffer, 0.1 M, pH 2.0, containing 2.5 M MgCl_2_. The number of times the membranes can be reused (three at most) is indicated in Table 4. Both membranes can be re-used for subsequent measures (as shown in Figure 7). However, although it was possible in all three case to obtain a good calibration curve, a decrease in calibration sensitivity is displayed both using the Pall-Biodyne membrane and the Immobilon one.

Lastly, the new biosensor was used to determine immunoglobulin G in human serum. Also the previously developed potentiometric immunosensor [7] had been used for this purpose. In the latter case, however, it was observed that the presence of urea in the serum could lead to non negligible interference [7]. On that occasion, it was attempted to overcome the problem by optimizing the number of washings carried out on the sensor membrane containing the immunocomplex before performing the enzymatic measurement. This optimization was moderately successful; nevertheless this procedure continued to be rather critical as one more or one less washing could lead respectively to an under - or an over-estimation of immunoglobulin G content. Using the new immunosensor, on the other hand, as was expected, in view of the extreme selectivity of both the immunological reaction and the biosensor used as transducer, urea interference is completely absent and the serum measurement can be carried out without any problem, as shown by the results of the measures displayed in Table 5.

In conclusion, using the present immunosensor it was easy to determine human immunoglobulin G in serum (Table 6) and in human milk; the latter results too are shown in Table 6. Lastly, results of recovery tests performed by the standard addition method both in serum and human milk are reported in Table 7.

As can be observed in Tables 6 and 7, analyses have been carried out both on sera and on milk samples with somewhat high concentrations of immunoglobulin G; in our case, for example, the test samples displayed a concentration of about 10^-4^ mM; usually concentrations of the order of about 10^-1^ - 10^-3^ mM are found in these human biological matrices, as reported in literature [11-13]; the determination of these samples thus necessarily required them to be diluted considerably so that final concentration would lie within the linear range of the method, as shown by the results reported in table 6. This operative choice was made: i) since it would make it possible to check if, when operating on increasingly diluted samples, the ability of the method to determine the total concentration of the analyte was not decreased; ii) in order to carry out enough repeatable measurements on samples that, being rich in proteins, are thus not fluid enough if undiluted. It was actually preferred to perform preventive dilution of the samples to render them more fluid and thus make the sampling operation more repeatable. On the other hand, it was important for the method to allow measurements also at very low concentrations of immunoglobulin G, even of the order of 10^-7^ mM. This is because, as reported in literature [14-19], in particular sera or milk samples, the values of immunoglobulin G do not exceed a concentration of 10^-4^ - 10^-5^ mM. Furthermore, it must be considered that the procedure used in the method also involves a further dilution of the sample, which in practice must often attain final concentrations of the order of 10^-7^ - 10^-8^ mM, that is, concentrations lying precisely in the linear range of the method described in the present research. It may therefore be stated that the proposed immunosensor method proved quite suitable for application to real biological samples both as far as a limit of detection of the order of 3×10^-11^ M is concerned, and with regard to the absence of any possible interference. From this standpoint it was found to be very suitable for this type of application, and not inferior to other now consolidated methods such as EIA type immunological methods [20], or nephelometric ones [21]. One of the advantages of the proposed biosensor method is in any case its simplicity, but above all its extreme cheapness. It may be applied, for instance, without the need for any ‘dedicated’ apparatus: all that is needed is a simple Clark type electrode coupled to an ordinary potentiostat, as well as a thermostated cell in which to perform the measurement. This means it can be used also in laboratories not possessing any particularly sophisticated equipment for this type of protein analysis; if necessary it can even be used to perform measurements “*in situ*”.

## 5. Conclusions

With the new amperometric immunosensor, which uses a tyrosinase biosensor as transducer, the sensitivity when the HIgG are measured is practically of the same order with both membranes (Immobilon or Pall-Biodyne); on the other hand, when the anti-HIgG are determined, the sensitivity is roughly twice as high when using the Pall-Biodyne membrane as with the Immobilon membrane. The linearity range extends over about two decades when measuring the HIgG and anti-HIgG both using Immobilon or Pall-Biodyne membranes. The limit of detection, of the order of 10^-8^ mM (i.e. about the same as is usually found in literature using other kinds of immunosystems) [22-33], is comparable both when the HIgG and the anti-HIgG are being determined. Compared with the previous potentiometric immunosensor [7], when either the HIgG or the anti-HIgG are measured, the limit of detection is practically of the same order, or only about half a decade higher; also the time of measurement is of the same order, but the new immunosensor is much more selective.

Lastly it became possible to determine immunoglobulin G in human milk and serum without the slightest interference by any urea contained in the human serum, also at low immunoglobulin G concentration levels. All this is due essentially to the principal novelty introduced in the construction of this new immunosensor. The novelty consists in using as transducer not an ordinary electrochemical sensor but an electrochemical biosensor, which gives to the immunosensor described herein a very high selectivity.

## Figures and Tables

**Table 1.** Analytical characterization of new immunosensor method for anti-HIgG determination, using two different membranes for antibody immobilization. Operating conditions: Buffer solution: Tris - HCl 0.1 M, pH 8.0; Incubation temperature 25 °C; Incubation time: 60 min.

Methods	Determination anti-HIgG by means of new immunosensor, that uses as transducer a tyrosinase enzyme electrode. Geometry of the test: competition for the HIgG, immobilized on the membrane, between the anti-HIgG conjugated with the alkaline phosphatase and not conjugated, both free in solution.	

Membrane Employed	Pall-Biodyne	Immobilon
Regression equation (Y= a.u., X= mM) level of confidence (1- α) = 0.95	Y = -2.01 (±0.63) logX-10.46 (±2.09) (n – *v*) = 8 ; (t = 2.31)	Y = -0.86 (±0.14) logX-4.66 (±1.84) (n – *v*) = 7 ; (t = 2.36)
Linear range (mM)	(0.52-37) × 10^-7^	(0.52 – 23) × 10^-7^
Correlation coefficient	0.9887	0.9872
Pooled SD%	5.5	5.8
Limit of detection (LOD) (mM)	0.25 × 10^-7^	0.35 × 10^-7^
Repeatability of the Measurement RSD %(n = 5)	6.6	7.2

**Table 2.** Analytical characterization of new immunosensor method for HIgG determination, using two different membranes for antibody immobilization. Operating conditions: Buffer solution: Tris - HCl 0.1 M, pH 8.0; Incubation temperature 25 °C; Incubation time: 60 min.

Methods	Determination HIgG by means of new immunosensor, that uses as transducer a tyrosinase enzyme electrode. Geometry of the test: competition for the free in solution anti-HIgG conjugated with the alkaline phosphatase, between the HIgG immobilized on the membrane and HIgG free in solution.	

Membrane Employed	Pall-Biodyne	Immobilon

Regression equation (Y=a.u., X= mM) level of confidence (1- α) = 0.95	Y = -1.19 (±0.26) log X-6.27 (±1.23) (n - *v*) = 6 ; (t = 2.45)	Y = -1.16 (±0.16) log X-6.40 (±1.54) (n - *v*) = 7 ; (t = 2.36)
Linear range (mM)	(0.52 - 37) × 10^-7^	(0.52 - 13) × 10^-7^
Correlation coefficient	0.9785	0.9770
Pooled SD%	5.8	6.1
Limit of detection (LOD) (mM)	0.25 × 10^-7^	0.26 × 10^-7^
Repeatability of the Measurement RSD (n = 5)	6.8	7.4

**Table 3.** Analytical characterization of biosensor method for anti-HIgG or HIgG determination, on the washing solution, using a simple tyrosinase biosensor for the measurement. Operating conditions: Buffer solution: Tris HCl 0.1 M, pH 8.0; Incubation temperature 25 °C; Incubation time: 60 min.

Methods	Determination of anti-HIgG by means of tyrosinase biosensor, on the washing solution, after immunocomplex formation on the immunosensor membrane		Determination of HIgG by means of tyrosinase biosensor, on the washing solution, after immunocomplex formation on the immunosensor membrane	

Membrane Employed	Pall-Biodyne	Immobilon	Pall-Biodyne	Immobilon

Regression equation (X= mM, Y= u.a) level of confidence (1- α) = 0.95	Y = 0.85 (±0.08) logX + 6.1 (±1.2) (n – *v*)= 8; (t = 2.31)	Y = 0.36 (±0.05) logX + 4.7 (±0.9) (n – *v*)= 7; (t = 2.36)	Y = 1.27 (±0.8) logX + 9.9 (±1.5) (n – *v*)= 8; (t = 2.31)	Y = 0.54 (±0.04) logX + 4.3 (±1.1) (n – *v*)= 7; (t = 2.36)
Linear range (mM)	(0.52 - 13) × 10^-7^	(0.52 – 13) × 10^-7^	(0.52 - 13) × 10^-7^	(0.52 – 10) × 10^-7^
Correlation coefficient	0.9756	0.9625	0.9755	0.9784
Pooled SD%	6.7	7.7	7.0	7.8
Limit of detection (LOD) (mM)	0.25 × 10^-7^	0.25 × 10^-8^	0.25 × 10^-8^	0.35 × 10^-8^
Repeatability of the Measurement RSD% (n=5)	6.9	7.3	6.8	7.2

**Table 4.** Reutilization of the new immunosensor for anti-HIgG analysis after washing with glycine solution 0.1 M, at pH = 2 and MgCl_2_ 2.5 M, using: **(a)** Pall-Biodyne membrane, **(b)** Immobilon membrane.

**(a)**		

Run of successive measures	Equation of the linear Regression (Y= a.u., X= mM of anti-HIgG)	Correlation coefficient

run 1	y = -2.0*1* (± 0.2) log x- 10.5 (±2.1)	r^2^ = 0.9887
run 2	y = -2.0*5* (± 0.4) log x- 8.5 (± 1.6)	r^2^ = 0.9786
run 3	y = -1.1*8* (± 0.3) log x- 7.8 (± 1.4)	r^2^ = 0.9377

**(b)**		

Run of successive measures	Equation of the linear regression (Y= a.u., X= mM of anti-HIgG)	Correlation coefficient

run 1	y = -0.8*6* (± 0.13) log x- 4.7 (± 1.8)	r^2^ = 0.9872
run 2	y = -0.7*7* (± 0.10) log x- 3.5 (± 1.2)	r^2^ = 0.9451
run 3	y = -0.4*8* (± 0.18) log x- 2.5 (± 1.6)	r^2^ = 0.9288

**Table 5.** Results of measures performed in human serum to verify possible urea interference. **(a)** = Signal (ppm O_2_), as a function of Log [HIgG], referring to serum solution at different dilutions; **(b)** = Signal (ppm O_2_), as a function of Log [HIgG], referring to serum solution at different dilutions (after addition to serum of 0.5 mL of urea solution 0.05 M); **(c)** = Signal (ppm O_2_), as a function of Log [HIgG], referring to washing solution from serum at different dilutions; **(d)** = Signal (ppm O_2_), as a function of Log [HIgG], referring to washing solution from serum at different dilutions (after addition to serum of 0.5 mL of urea solution 0.05 M).

Human serum	n= 5 RSD% ≤ 5 **(a)**	n= 5 RSD% ≤ 5 **(b)**	$\frac{\left(\text{b}-\text{a}\right)}{\text{a}}\times 100$	n= 5 RSD% ≤ 6 **(c)**	n= 5 RSD% ≤ 6 **(d)**	$\frac{\left(\text{d}-\text{c}\right)}{\text{c}}\times 100$

Diluted 1:100,000	2.44*6*	2.45*2*	+0.2*4*	0.17*6*	0.16*8*	-4.5*5*
Diluted 1:10,000	1.45*8*	1.44*2*	-1.1*0*	0.32*7*	0.34*8*	-6.4*2*
Diluted 1:1,000	0.54*6*	0.49*0*	-4.8*2*	0.57*8*	0.58*6*	-1.38
Diluted 1:100	0.16*2*	0.16*5*	+1.8*5*	0.89*2*	0.89*9*	-0.7*8*

**Table 6.** Determination by new immunosensor of immunoglobulin G in human serum and human milk (at different dilutions).

Biological Matrix	Dilution	Final concentration immunoglobulin G in the diluted sample (mM) n=5; RSD%≤ 5	Found concentration immunoglobulin G in the undiluted sample (mM) n= 5; RSD%≤ 5

Human Serum	1:10,000	2.3*0* × 10^-8^	2.3*0* × 10^-4^
Human Serum	1:1,000	1.9*5* × 10^-7^	1.9*5* × 10^-4^
Human Serum	1:100	2.4*8* × 10^-6^	2.4*8* × 10^-4^

Human Milk	1:1,000	2.0*3* × 10^-7^	2.0*3* × 10^-4^
Human Milk	1:100	1.8*2* × 10^-6^	1.8*2* × 10^-4^

**Table 7.** Recovery tests by new immunosensor of added immunoglobulin G in human serum and human milk (at different dilutions).

Biologica l Matrix	Dilution	Final concentration Immunoglobulin G in the diluted sample (mM) (n=5); RSD%≤ 5	Added immunoglobulin G concentration (mM)	Experimental immunoglobulin G concentration (mM) (n= 5); RSD%≤ 5	Recovery % immunoglobulin G concentration in the diluted sample

Human Serum	1:1,000	1.9*5* × 10^-7^	3.5*0* × 10^-7^	5.5*8* × 10^-7^	102.4
Human Serum	1:100	24.*8* × 10^-7^	3.5*0* × 10^-7^	29.2*1* × 10^-7^	103.2

Human Milk	1:1,000	2.0*3* × 10^-7^	12.*7* × 10^-7^	14.*8* × 10^-7^	99.5
Human Milk	1:100	13.*2* × 10^-7^	12.*7* × 10^-7^	25.*2* × 10^-7^	97.3
